# Long-term results of the modified Dunn procedure in slipped capital femoral epiphysis: a minimum of ten-year follow-up

**DOI:** 10.1302/2633-1462.710.BJO-2026-0076.R1

**Published:** 2026-10-12

**Authors:** Alessandro Aprato, Andrea Audisio, Samuele Grilli, Matteo Giachino, Virginia Reinaudo, Salvatore Risitano, Reinhold Ganz, Alessandro Masse'

**Affiliations:** 1 University of Turin, Turin, Italy; 2 Regina Margherita Children's Hospital, Città della Salute e della Scienza, Turin, Italy; 3 CTO Hospital, Città della Salute e della Scienza, Turin, Italy; 4 University of Bern, Bern, Switzerland

**Keywords:** Slipped capital femoral epiphysis, modified Dunn Procedure, Hip, Patient reported outcome measures, Proximal femoral deformity, Conservative hip surgery, Osteotomy, modified Dunn procedure, Slipped capital femoral epiphysis (SCFE), hips, Functional outcomes, femoroacetabular impingement, avascular necrosis, total hip arthroplasty (THA), Patient-reported outcome measures (PROMs), early osteoarthritis, proximal femoral deformity

## Abstract

**Aims:**

In situ fixation for slipped capital femoral epiphysis (SCFE) prevents further displacement, but fails to correct the proximal femoral deformity, which may lead to femoroacetabular impingement (FAI) and early osteoarthritis (OA). The modified Dunn procedure (MDP) restores proximal femoral anatomy with the aim of improving long-term joint preservation. However, evidence beyond mid-term follow-up remains limited. This study evaluates the ten-year survivorship, functional outcomes, and radiological correction following MDP.

**Methods:**

A retrospective single-centre review was performed of patients treated with the MDP between 2007 and 2014 for stable or unstable SCFE of any severity. Patients with preoperative avascular necrosis (AVN) were excluded. Primary outcomes were ten-year survivorship free from conversion to total hip arthroplasty (THA) and from radiological OA progression (≥ 1 Tönnis grade). Secondary outcomes included patient-reported outcome measures, hip range of motion, clinical signs of FAI, and correction of the Southwick and α angles. Kaplan-Meier survival analyses were performed and stratified by slip severity.

**Results:**

A total of 35 hips (34 patients) completed ≥ ten years of follow-up (163 months (SD 34)). Ten-year THA conversion-free survivorship was 88.6%, decreasing from 100% in mild slips to 86.4% in moderate and 83.3% in severe slips. Ten-year OA progression–free survivorship was 82.9%, with rates of 100%, 81.8%, and 66.7% for mild, moderate, and severe slips, respectively. The mean ten-year functional scores were high (modified Harris Hip Score 88.9 (SD 4.4); University of California, Los Angeles Activity Scale 8.9 (SD 1.6); and Merle d’Aubigné-Postel 17.7 (SD 0.9)). Substantial radiological correction was observed, with the Southwick angle improving from 45.0° to 13.1° and the α angle from 85.8° to 50.9°. No postoperative AVN occurred.

**Conclusion:**

At ten-year follow-up, the MDP achieved excellent functional outcomes and effective deformity correction in SCFE, with high survivorship in mild and moderate slips. However, progression of hip OA was less favourable in severe slips.

Cite this article: *Bone Jt Open* 2026;7(10):1295–1303.

## Introduction

Slipped capital femoral epiphysis (SCFE) is a common hip disease during childhood and adolescence affecting about 10/100,000 patients, with a range 0.33 to 24/100,000, depending on sex and ethnicity, and with a peak incidence between aged ten and 12 years.^1^ In situ percutaneous fixation with cannulated screws or Kirschner (K)-wires is a suitable option to prevent further displacement.^2^ While a minimally invasive procedure with low perioperative complications, it does not address the proximal femoral deformity which can lead to femoroacetabular impingement (FAI) and early-onset osteoarthritis (OA).^3-6^ Anatomical correction of the slip angle seeks to restore physiological hip morphology and may mitigate the mechanical factors associated with early osteoarthritis. Dunn first introduced the subcapital realignment technique for the treatment of slipped capital femoral epiphysis, providing a means to directly correct the femoral deformity and restore proximal femoral anatomy.^7^ However, the original procedure carried a substantial risk of avascular necrosis due to limited protection of the epiphyseal blood supply.^8^ Ganz et al^9,10^ modified the original procedure by developing the extended retinacular flaps, preserving the epiphyseal vascularization while achieving anatomical realignment.^11^ A low rate of AVN is reported at the inventor’s institution (2%).^12^ While encouraging results have been reported, including the series by Ziebarth et al^13^ evidence at long follow-up remains limited, particularly regarding osteoarthritis progression and functional outcomes.^14-17^ In addition, most available data derive from developer centres, and the reproducibility of these findings in independent series is not yet fully established. In this context, the present study provides ten-year survivorship and functional outcomes from a non-developer centre. We retrospectively reviewed patients treated by the modified Dunn procedure (MDP)^11^ with a minimum ten-year follow-up. The aims of our study were: 1) ten-year survival rate defining failure as conversion to total hip arthroplasty (THA); 2) OA progression-free ten-year survival rate defining failure as progression of OA by at least grade according to Tönnis classification;^18^ 3) ten-year functional results evaluated through clinical signs of FAI, hip range of motion (ROM), modified Harris Hip Score (mHHS),^19^ University of California, Los Angeles Activity Scale (UCLA) score,^20^ and Merle d’Aubigné-Postel score;^21^ and 4) radiological outcomes assessed through Southwick and α angle correction.

## Methods

All patients presenting to our institution between January 2007 and December 2014 with stable or unstable SCFE, regardless of slip severity, and treated with the MDP were retrospectively included in this study. Exclusion criteria were patients with established AVN before the index procedure and those with minimum slip treated by in situ fixation alone. All surgeries were performed by one senior hip surgeon (AM) with extensive experience in hip preservation surgery, following the technique originally described by Ganz et al.^11^ Informed consent was obtained from all patients and their legal guardians. The study protocol received a waiver from the local ethics committee.

The primary outcome was the ten-year survival rate, with failure defined as conversion to THA; although this represents a single-surgeon series, the decision to proceed to THA was made during follow-up by different members of the treating team and was not solely dependent on the operating surgeon. The secondary outcome was a OA progression-free ten-year survival rate, with failure defined as progression of OA by at least one Tönnis grade. The tertiary outcome was ten-year functional result that assessed exclusively in hips that had not undergone conversion to THA; hips converted to arthroplasty were therefore excluded from this analysis. Patient-reported outcome measures (PROMs) were collected using the mHHS, the UCLA Activity score, and the Merle d’Aubigné-Postel Score. Functional outcomes were classified using the mHHS as excellent ( ≥ 81), good (72 to 80), fair (63 to 71), or poor ( ≤ 62). Clinical evaluation included assessment of hip ROM, the impingement test, and the Drehmann sign.^22^ Hip ROM was measured with a goniometer; internal and external rotation were assessed at 90° of flexion, or, in cases where 90° was not attainable, at the maximum flexion achievable by the patient. The quaternary outcome was the radiological analysis of proximal femoral morphology, comparing preoperative and postoperative anteroposterior (AP) pelvis and frog-leg lateral views. radiological parameters included the Southwick angle, the α angle, and their degree of correction achieved postoperatively, inter- and intraobserver reliability for radiological measurements was not formally evaluated.

### Statistical analysis

Ten-year survival was estimated using the Kaplan-Meier method, with THA conversion or OA progression by ≥ 1 Tönnis grade as events. Survival curves were stratified by clinical variables, including slip severity and Loder classification, and compared using the log-rank test. Continuous variables (demographics, hip ROM, and PROMs) were summarized as mean (SD) and compared using independent-samples or paired *t*-tests as appropriate. Repeated measures were analyzed with analysis of variance (ANOVA) for continuous variables or Cochran Q test for categorical variables, with post-hoc pairwise comparisons using McNemar test with Bonferroni correction. Statistical significance was set at p < 0.05. Analyses were performed using SPSS v. 28.0 (IBM, USA).

## Results

In this study, 35 hips (34 patients) were eligible for a minimum ten-year complete clinical and radiological follow-up. One patient had bilateral involvement at presentation, the two procedures were carried out within a four-month interval (Figure 1). According to Loder classification, six hips (17.1%) presented as unstable SCFE.^23^ According to the Southwick classification, seven hips (20%) presented with a mild slip, 22 hips with a moderate slip (62.9%) and six hips with a severe slip (17.1%).^24^ Demographic and clinical data are summarized in Table I.

**Fig. 1:**
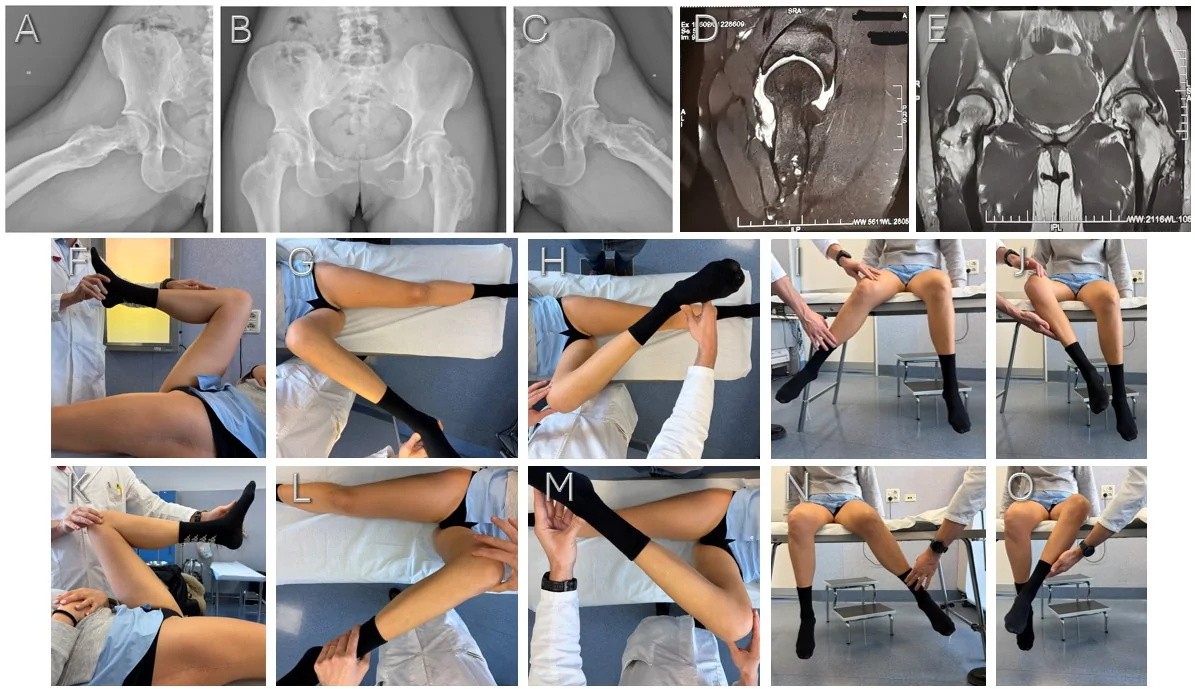
(a to c) Bilateral slipped capital femoral epiphysis treated with modified Dunn procedure and subsequent hardware removal. (f to j) Aanteroposterior pelvis and frog-leg view radiographs at 18-year follow-up. MRI at 18year follow-up. (d, e) Right hip clinical examination at 18-year follow-up showing complete range of motion (ROM) (flexion 120°, internal rotation 30°, external rotation 45°). (k to o) Left hip clinical examination at 18-year follow-up showing complete ROM (flexion 120°, internal rotation 30°, external rotation 45°).

**Table I.** Demographic and clinical data.

**Variable**	**Data**
Hips/patients, n	35/34
Mean age at operation, yrs (SD)	13.2 (2)
**Sex, n (%)**	
Male	22 (62.9)
Female	12 (37.1)
Mean BMI, kg/m² (SD)	24.1 (5.4)
**Side, n (%)**	
Right	16 (45.8)
Left	19 (54.2)
Mean time to surgery, days (SD)	217 (194)
**SCFE onset, n (%)**	
Acute	4 (11.4)
Chronic	29 (82.9)
Acute or chronic	2 (5.7)
**SCFE severity (Southwick), n (%)**	
Mild	7 (20.0)
Moderate	22 (62.9)
Severe	6 (17.1)
**SCFE stability (Loder), n (%)**	
Stable	29 (82.9)
Unstable	6 (17.1)

SCFE, slipped capital femoral epiphysis.

The mean follow-up was 163 months (SD 34; 20 to 218). THA conversion-free ten-year survival rate according to Kaplan-Meier was 88.6% (31/35 hips); four patients underwent conversion to THA (after four, five, eight, and nine years after MDP, respectively). Stratifying by slip severity according to the Southwick angle, the THA conversion-free ten-year survival rate was 100% for mild SCFE (7/7), 86.4% for moderate SCFE (19/22) and 83.3% for severe SCFE (5/6) (Figure 2). Overall, the OA progression-free ten-year survival rate according to Kaplan-Meier was 82.9% (29/35 hips); stratified by slip severity, the OA-free ten-year survival rate was 100% for mild slips (7/7), 81.8% for moderate slips (18/22), and 66.7% for severe slips (4/6) (Figure 3). No statistically significant differences in ten-year survival were observed when stratifying the cohort by Loder classification (Figure 4) or by time from symptom onset to surgery. At ten-year follow-up, 29 hips were Tönnis 0 (82.9%), one hip Tönnis 2 (2.9%), and five hips Tönnis 3 (14.3%), four of which underwent THA conversion.

**Fig. 2:**
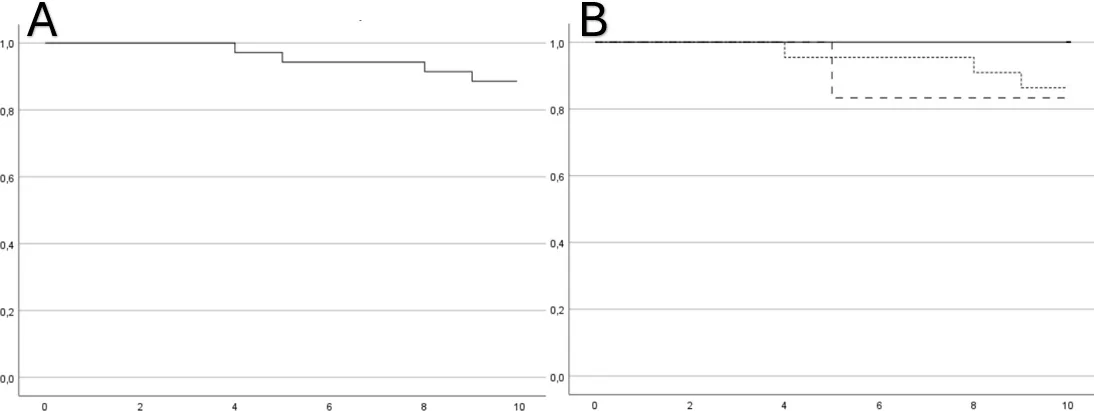
a) Kaplan-Meier curve showing total hip arthroplasty (THA) conversion-free survival at ten-year follow-up. b) Kaplan-Meier curve compared THA conversion-free survival at ten-year follow-up according to slip severity: Plain line, mild slip; small dotted line, moderate slip; and large dotted line, severe slip. The y-axis shows survival probability and the x-axis shows time in years.

**Fig. 3:**
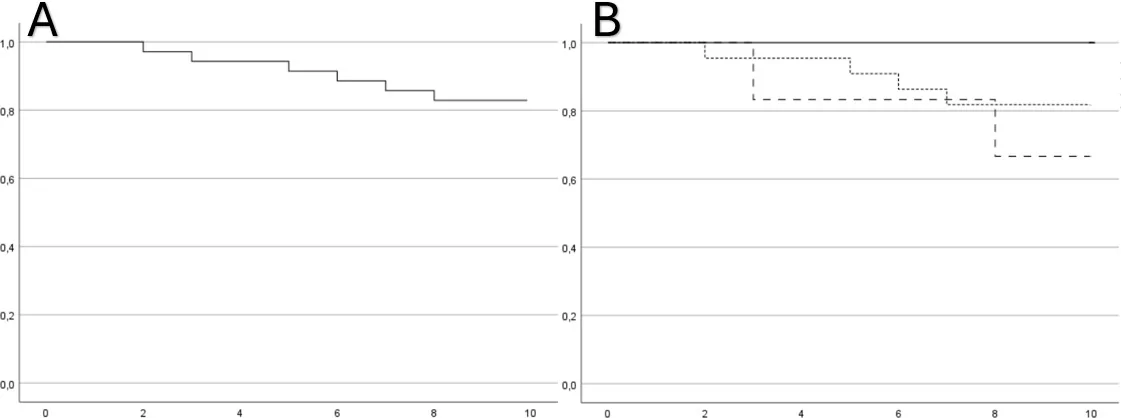
a) Kaplan-Meier curve showing osteoarthritis (OA) progression-free survival at ten-year follow-up. b) Kaplan-Meier curve compared OA progression-free survival at ten-year follow-up according to slip severity: Plain line, mild slip; small dotted line, moderate slip; and large dotted line, severe slip. The y-axis shows survival probability and the x-axis shows time in years.

**Fig. 4:**
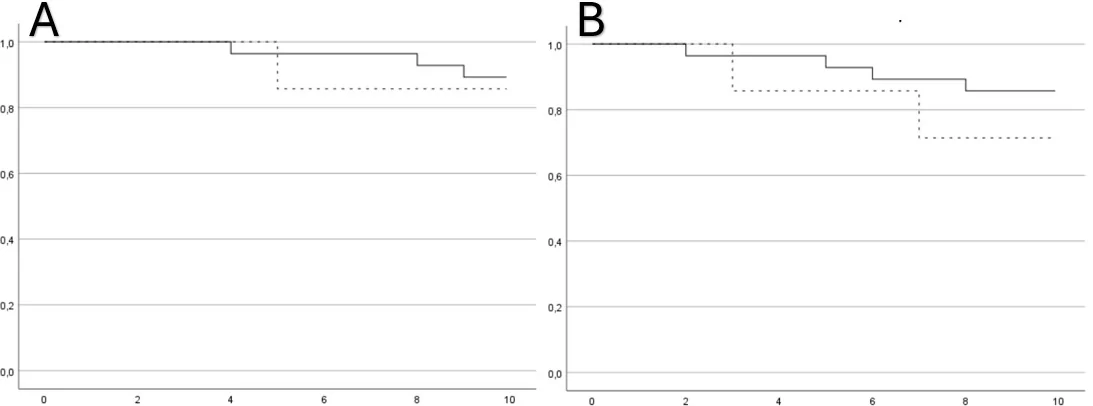
a) Kaplan-Meier curve comparing total hip arthroplasty conversion-free survival at ten-year follow-up according to Loder classification: Plain line, Loder I (stable slips); large dotted line, Loder II (unstable slips). b) Kaplan-Meier curve compared osteoarthritis progression-free survival at ten-year follow-up according to Loder classification: Plain line, Loder I (stable slips); large dotted line, Loder II (unstable slips). The y-axis shows survival probability and the x-axis shows time in years.

Functional outcomes were available for 31 hips that had not undergone conversion to THA at ten-year follow-up. The mHHS was 88.9 (SD 4.4; 77 to 91); all hips had excellent functional results except three reporting good results. At the ten-year follow-up the UCLA score was 8.9 (SD 1.6; 5 to 10) with 13 patients (14 hips, 41%) reporting an excellent result (10/10). The Merle d’Aubigné-Postel score was 17.7 (SD 0.9; 15 to 18). postoperative hip ROM improved in all patients: at ten-year follow-up the mean internal rotation was 27.8 (SD 5.0°; 15 to 30) and the mean hip flexion was 113.5 (SD 14.1°; 60 to 120). The prevalence of a positive impingement test decreased from 80% (28/35 hips) preoperative to 8.6% (3/35) postoperatively; similarly, the prevalence of a positive Drehmann sign decreased from 62.8% (22/35) preoperative to 5.7% (2/35) postoperatively. A total of 11 patients (12 hips) underwent subsequent surgery for screws removal at a mean interval of 26 months (13 to 37) from MDP. No additional surgical procedures, including hip arthroscopy, were performed during the follow-up period. The Southwick angle on frog-leg view was 45.0 (SD 14.8°; 19 to 71.3) preoperatively and 13.1 (SD 8.1°) postoperative resulting in a correction of 31.9 (SD 12.0°). The α angle was 85.8 (SD 6.5°; 61 to 95) preoperatively and 50.9 (SD 4.8°) postoperatively, resulting in a correction of 34.9 (SD 7.2°). Functional and radiological outcomes at ten-year follow-up are summarized in Table II.

**Table II.** Summary of preoperative and postoperative functional and radiological outcomes

				p-value	
Variable	Preoperative	Two-yr follow-up	Ten-yr follow-up	Overall comparison	Pairwise comparison
Mean Southwick angle, ° (SD)	45.0 (18.4)	13.1 (8.9)	-	< 0.001*	-
Mean α angle, ° (SD)	87.2 (15.1)	50.9 (4.8(	50.4 (4.6)	< 0.001†	Pre-op vs two yrs: < 0.001* Pre-op vs ten yrs: < 0.001* Two yrs vs ten yrs: 0.930*
**Tonnis grade, n**					
0	35	34	29		
1	-	-	-		
2	-	-	1		
3	-	1	5‡		
Mean hip internal rotation, ° (SD)	2.9 (8.7)	28.1 (4.3)	27.5 (8.1)	< 0.001†	Pre-op vs two yrs: < 0.001* Pre-op vs ten yrs: < 0.001* Two yrs vs ten yrsr: 0.700*
Mean hip flexion, ° (SD)	69.2 (23)	115 (9.4°)	113 (15.4)	< 0.001†	Pre-op vs two yrs: < 0.001* Pre-op vs ten yrs: < 0.001* Two yrs vs ten yrs: 0.510*
Impingement test, n (%)	28/35 (80.0)	3/35 (8.6)	3/31 (9.6)	< 0.001§	Pre-op vs two yrs: < 0.001¶ Pre-op vs ten yrs: < 0.001¶ Two yrs vs ten yrs: 1.000¶
Drehmann sign, n (%)	22/35 (62.8)	2/35 (5.7)	2/31 (6.4)	< 0.001§	Pre-op vs two yrs: < 0.001¶ Pre-op vs ten yrs: < 0.001¶ Two yrs vs ten yrs: 1.000¶
**Mean score (SD)**					
modified Harris Hip	-	89.1 (3.5)	88.9 (4.3)	0.830*	-
UCLA Activity score	-	9.1 (1.1)	8.9 (1.6)	0.550*	-
Merle D'Aubigné	-	17.74 (0.8)	17.68 (0.9)	0.810*	-

* Paired *t*-test.

† Analysis of variance test.

‡ Four hips were converted to total hip arthroplasty before ten-year follow-up.

§ Cochran Q test.

¶ McNemar test with Bonferroni adjustment.

UCLA, University of California, Los Angeles Activity Scale.

## Discussion

In situ pinning has traditionally been regarded as the standard treatment for SCFE, regardless of slip severity. Nonetheless, some studies have reported degenerative changes in a significant proportion of affected hips, with a reported conversion rate to THA ranging between 16% and 40%.^6,25^ Improved understanding of femoral head vascular anatomy has enabled the development of surgical techniques aimed at achieving safe subcapital anatomical realignment of the slipped epiphysis.^10,26^ The Dunn procedure, as modified by Ganz et al,^11^ was developed to restore the native proximal femoral anatomy, correct anterior metaphyseal overhang, and thereby address FAI which may contribute to early cartilage damage and subsequent hip OA. Early concerns regarding its effect on femoral head vascularity and the risk of avascular necrosis have been mitigated by reports indicating that, when performed by experienced surgeons following the described technique, the procedure can be both safe and effective in preserving femoral head blood supply.^13-17^

At ten years, survivorship in our cohort was 88.6% when THA conversion was considered the endpoint and 82.9% when OA progression was used. Stratified by slip severity, mild slips showed the most favourable outcomes, with 100% THA conversion-free and OA-free survival, followed by moderate (86.4% and 81.8%) and severe slips (83.3% and 66.7%). Although survivorship appeared lower in moderate and severe slips, these differences were not statistically significant. The relatively small sample size, particularly in the severe slip subgroup, reflects the low incidence of the condition and limits the statistical power of subgroup analyses. Ziebarth et al^27^ reported a ten-year survival rate of 93%. Differences in survival rates between the two series may be partly attributable to differences in slip severity distribution, with a lower proportion of mild slips in our cohort (12%) compared with their study (23%).

Delayed diagnosis remains common in SCFE, largely due to initially vague symptoms, early assessment by non-orthopaedic providers, and the need for referral to specialized centres with appropriate surgical expertise.^28,29^ In our series, the mean interval between symptom onset and surgery exceeded 210 days, with five patients referred to our institution after nearly two years of hip pain. The correlation between delayed diagnosis and poor outcomes remains controversial in the literature; some authors have suggested that late presentation is associated with more severe slips and, consequently, greater long-term functional limitations.^30^ However, in our study, there was no statistically significant difference in either ten-year survival rate or functional outcomes according to the time between symptom onset and the MDP.

Several studies have suggested that unstable SCFE, as defined by the Loder classification, is associated with an increased risk of AVN and potentially poorer clinical outcomes.^31-33^ However, in our cohort, there was no statistically significant difference in the ten-year survival rate between stable and unstable SCFE (p = 0.280). We believe that the data reported in the literature may be influenced by the high incidence of AVN observed in cases of unstable SCFE treated with in situ fixation alone, where acute displacement may subject the retinacular vessels to traction or compression, potentially compromising femoral head perfusion. In contrast, the use of extended retinacular flaps in the MDP allows careful handling and decompression of the vessels, providing an opportunity for femoral head reperfusion. These considerations suggest that the MDP may offer a safer approach for unstable SCFE, potentially reducing the risk of AVN compared with in situ fixation.

The prevalence of a positive Drehmann sign decreased from 65% preoperatively to 6% following the MDP. In our series, persistence of a positive Drehmann sign was associated with poorer long-term functional outcomes: one patient developed early OA with moderate functional limitation but declined further surgery, while the other showed radiological signs of OA at 13 years but reported only tolerable pain and declined THA conversion.

Functional outcomes at long-term follow-up were excellent in the majority of patients. At ten-year follow-up, the mean mHHS was 88.9 points (maximum achievable score 91). At latest follow-up, only three patients reported mild, occasional pain without any limitation in daily activities, and another three patients experienced moderate pain requiring intermittent use of nonsteroidal anti-inflammatory drugs (NSAIDs), occurring 12, 13, and 15 years after the MDP. At ten-year follow-up, the mean UCLA Activity score was 8.9, with 14 patients regularly participating in high-impact sports (UCLA = 10), whereas five patients were limited to low- or moderate-intensity activities (UCLA < 8). Published studies on in situ fixation for SCFE have reported significantly lower long-term functional outcomes, with mean mHHS and UCLA scores typically below 80 and 8, respectively, and with nearly two-thirds of patients engaging only in low-impact activities such as swimming or cycling.^34^ This finding suggests that preventing further slippage without addressing the underlying FAI may lead to reduced activity levels due to functional limitations or pain from the residual deformity. The mean Merle d’Aubigné-Postel Score was 17.7, comparable to Ziebarth et al’s^27^ results at ten-year follow-up. Functional outcomes were assessed only in hips that had not undergone conversion to THA, in order to characterize the functional results of joint-preserving treatment. However, this approach may introduce selection bias and potentially overestimate overall functional outcomes.

We retrospectively analyzed all cases of failure. In our series, four hips were converted to THA. One of these hips developed septic arthritis several years after the MDP, leading to secondary OA; although further surgical intervention was initially postponed, conversion to THA became necessary nine years after MDP (Figure 5). The remaining three hips failed due to progressive OA, requiring THA at four, five, and eightyears after MDP, respectively. One hip developed chondrolysis and subsequently progressed to OA with functional limitations. However, at the latest follow-up, the patient had still declined conversion to THA (Figure 6). Importantly, no hips in our series showed radiological evidence of AVN at any stage of follow-up.

**Fig. 5:**
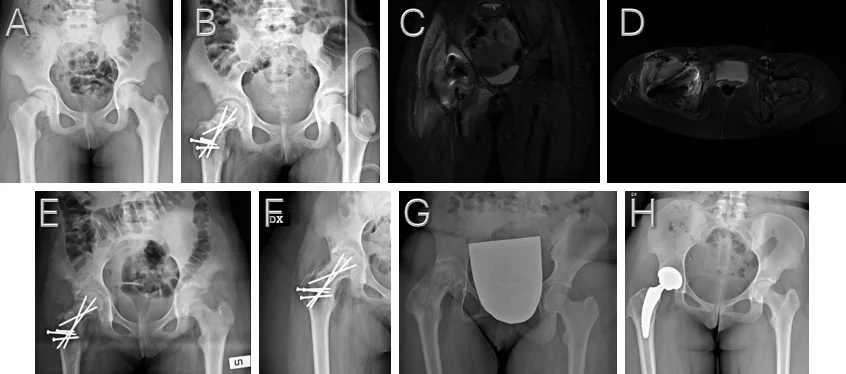
Hip failure due to septic arthritis and subsequent osteoarthritis a) Anteroposterior pelvis at slipped capital femoral epiphysis diagnosis. b) modified Dunn procedure (MDP) postoperative anteroposterior (AP) pelvis. c, d) Pelvis MRI showing altered intensity signal in the soft-tissue of the right hemipelvis and intra-articular fluid, patient underwent arthroscopic irrigation and debridement and antibiotics therapy. e) AP pelvis radiograph at arthroscopic irrigation. f) Radiograph showing OA progression after septic arthritis resolution. g) Hardware removal at fve years from MDP. h) Conversion to THA at nine years from MDP.

**Fig. 6:**
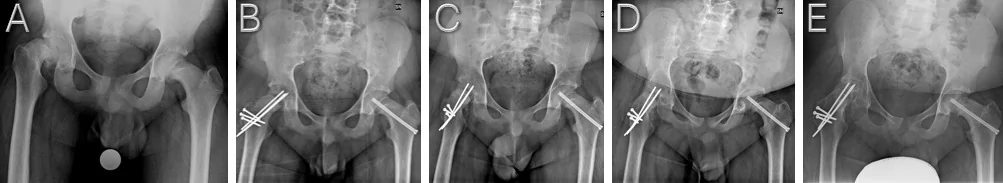
Hip failure due to chondrolysis. a) Anteroposterior (AP) pelvis at SCFE diagnosis. b) modified Dunn procedure postoperative AP pelvis.c) One-month AP pelvis showing narrowing of joint line in absence of femoral head collapse. d) Three-month AP pelvis showing further narrowing of the joint space.e) Progression to hip osteoarthritis at one year from MDP.

This study represents a single-centre, single-surgeon series, which may introduce surgeon-related bias and limit generalizability. The favourable outcomes observed are therefore likely influenced, at least in part, by surgical experience and strict adherence to the described technique. However, this design also ensures procedural consistency and reduces variability related to technique.

This study has several limitations. First, its retrospective design and the absence of a control group treated with alternative surgical techniques limit the ability to perform direct comparisons. Consequently, our survival rates and functional outcomes can only be compared with previously published data from other series. Second, the future long-term outcomes of the MDP at our institution may not be directly comparable with the current cohort due to differences in patient selection. In the present study, mild SCFEs demonstrated the best results; however, we now reserve the MDP for moderate and severe slips, while all mild cases are treated with in situ pinning combined with arthroscopic trimming (ISPAT). Given that a previous study at our institution demonstrated a comparable degree of correction of FAI parameters in mild SCFE treated either with the MDP or ISPAT, we now prefer the less invasive approach for these cases. This strategy allows effective management of the deformity while minimizing surgical morbidity and preserving excellent short-term functional outcomes.^35^ Third, although AVN represents the most serious complication of the MDP, we did not perform systematic MRI screening to assess femoral head viability. All patients were considered free of AVN, as no radiological signs of necrosis were observed on plain radiographs at long-term follow-up. In the end, the PROMs used in this study may be subject to ceiling effects in a young and active population. More recently, developed hip-specific instruments, such as the 12-item international Hip Outcome Tool (iHOT-12) or iHOT-33, may provide greater sensitivity in this setting and could be considered in future studies.

In conclusion, restoration of proximal femoral anatomy remains an important goal in the treatment of SCFE, as it may mitigate FAI and the subsequent risk of chondral damage and early hip OA in young and active patients. The MDP represents a viable surgical option to achieve anatomical realignment while maintaining an acceptable risk profile when performed by experienced surgeons in accordance with the described technique. In our series, at ten-year follow-up, hip survival exceeded 88%, with the majority of patients reporting minimal pain, favourable functional outcomes, and a return to an active lifestyle.

**Take home message**

- At a minimum ten-year follow-up, the modified Dunn procedure achieves reliable anatomical correction, excellent function, and low complication rates in slipped capital femoral epiphysis, with durable hip preservation in mild and moderate slips, while severe slips remain at increased risk for long-term osteoarthritis.

## Data Availability

The datasets generated and analyzed in the current study are not publicly available due to data protection regulations. Access to data is limited to the researchers who have obtained permission for data processing. Further inquiries can be made to the corresponding author.
